# Visual and AI-Based Assessment of COVID-19 Pneumonia: Practicability and Reproducibility of an Established Semi-Quantitative Chest CT Scoring System

**DOI:** 10.3390/diagnostics15161987

**Published:** 2025-08-08

**Authors:** Eugen Neumann, Anna Movlilishvili, Simon T. Scherfeld, Lubana Al Haj Hossen, Ulf Titze, Johann P. Addicks, Michel Eisenblätter, Anna J. Höink

**Affiliations:** 1 Department of Diagnostic and Interventional Radiology, Klinikum Lippe, Medical School and University Medical Center OWL, Bielefeld University, 32756 Detmold, Germany; eugen.neumann@klinikum-lippe.de (E.N.);; 2 Department of Pathology, Klinikum Lippe, Medical School and University Medical Center OWL, Bielefeld University, 32756 Detmold, Germany

**Keywords:** CT scoring system, COVID-19 pneumonia, AI-based assessment, inter-reader agreement, chest CT

## Abstract

**Background/Objectives**: To determine the inter-rater agreement of visual and AI-based assessments of a renowned semi-quantitative chest CT scoring system (Pan-score) used to evaluate the severity of pulmonary involvement (e.g., ground-glass opacities, consolidations) in patients suffering from COVID-19. **Methods**: This retrospective study includes patients with PCR-confirmed COVID-19, who received a chest CT scan (not more than three days prior to or after the positive PCR test) between 21 March 2020 and 30 December 2022. The five lung lobes were scored separately on a scale from 0 (no pulmonary involvement) to 5 (>75% pulmonary involvement) by a radiology specialist, an experienced assistant physician, a medical student, and a dedicated AI-based software tool for chest CT. Weighted Cohen’s κ values were calculated to assess the reliability of agreement between the different readers. **Results**: A total of 569 consecutive patients (381 males [67.0%], 188 females [33.0%]; mean age 68.8 years) with confirmed COVID-19 were evaluated. All of them received at least one chest CT scan. There was a significant difference (*p* < 0.001) between the mean Pan-score evaluated by the three human readers (9.35 ± 6.03) and the score computed fully automatically by the software (10.44 ± 5.10). However, the inter-rater agreement both between the three different human readers and between the human readers and the AI was high throughout, with κ values of 0.71–0.86 and 0.83, respectively. The slice thickness of the reconstructed CT images did not have an impact on the inter-rater agreement, but the total score was significantly higher when the images were acquired following the administration of i. v. contrast media. **Conclusions**: The evaluated chest CT scoring system is user-friendly due to its simplicity, though it is generally prone to inaccuracies, since the estimation of the extent of pulmonary involvement is quite subjective. Nevertheless, the inter-rater agreement was high throughout, both between the differently experienced human readers and between the human readers and the AI software. In summary, the Pan-score seems to be a reliable approach to estimate the extent of pulmonary involvement in patients suffering from COVID-19.

## 1. Introduction

In March 2020, the coronavirus disease 2019 (COVID-19) caused by the severe acute respiratory syndrome coronavirus 2 (SARS-CoV-2) was declared a pandemic by the World Health Organisation (WHO). By the end of February 2025, more than 777,500,000 cases of COVID-19 and around 7,000,000 deaths had been reported to the WHO worldwide. Even though mortality rates have been steadily reduced over the course of the pandemic, thanks to vaccinations as well as hygiene and social measures, COVID-19 must still be classified as endemic in Germany. The now high level of immunity in the population has led to fewer severe cases and long-term consequences. However, it is assumed that there will be further waves of spread in the future, which will then primarily affect older and previously ill people [1].

Since the beginning of the pandemic, X-ray and, in particular, computed tomography (CT) examinations of the thorax have been of great importance, not only in the assessment of the course of a COVID-19 infection and for the detection of complications but also for the confirmation of a suspected diagnosis, for example, in the event of a negative polymerase chain reaction (PCR) test with a high pre-test probability or COVID-19-typical symptoms [2].

COVID-19 pneumonia typically manifests itself in the form of ground-glass opacities (GGO), which are multifocal and usually bilateral, have a patchy or map-like morphology, and primarily affect the periphery and the posterobasal sections of the lungs. Consolidations and a crazy-paving pattern can be observed less frequently or in advanced stages of the disease [3]. Moreover, interlobular septal thickening, vascular enlargement, and a halo sign are common parenchymal changes, described in many studies in connection with COVID-19 pneumonia [4].

It has already been shown that the quantitative assessment of changes in the lung parenchyma is an important factor in estimating the prognosis of COVID-19 [5]. One example is the semi-quantitative CT score proposed by Pan et al., in which each lung lobe is assessed individually based on the extent of its involvement (0, no involvement, to 5, >75% of the parenchyma involved). A total score, i.e., the sum of the scores of the five lung lobes, of ≥18 is associated with a significantly higher risk of a fatal course of COVID-19 pneumonia [6,7]. For this score, however, the type of parenchymal changes is irrelevant; scores are based on the extent of the changes only.

The aim of this study was to evaluate the score proposed by Pan et al., with regard to its practicability, accuracy, and reproducibility, using a patient cohort, which is considerably larger than the cohorts of previous studies in order to obtain more reliable results. The objective was to investigate the consistency of this purely visual and therefore subjective score across readers with different diagnostic experience. In addition, it should be explored whether a software based on artificial intelligence (AI) would enable a reliable application of the CT score and produce comparable results.

Our hypotheses were that the more experienced the readers, the lower the inter-observer variability, and that the AI-based software results do not differ significantly from those of human readers.

## 2. Materials and Methods

This retrospective study includes 569 consecutive patients (381 males [67.0%], 188 females [33.0%]; mean age 68.8 years) with polymerase chain reaction (PCR)-confirmed COVID-19, who received a chest CT scan at our hospital between 21 March 2020 and 30 December 2022. In March 2020, the first case of COVID-19 was confirmed in our district. This means that the period chosen allowed for the largest possible patient cohort to be formed at the time the data analysis began.

The precise inclusion criteria were (a) positive PCR test for COVID-19, (b) chest CT scan performed not more than three days prior to or three days after the positive PCR test, and (c) age ≥18 years. In total, 38 of the original 607 patients had to be excluded due to incomplete CT data sets, motion/breathing artefacts, prior extensive lung surgery, or other technical difficulties that made AI-based evaluation impossible. The study was approved by the local ethics committee (Ärztekammer Westfalen-Lippe and University of Münster, both located in Münster, Germany) and conducted according to the Declaration of Helsinki.

The respective CT images were selected from the hospital’s picture archiving and communication system (PACS; Centricity^TM^ Universal Viewer, Version 6.0, GE HealthCare Technologies Inc., Chicago, IL, USA) and collected in a separate list within the radiological information system (RIS; Centricity^TM^ RIS-i7, Version 7.0.4.4, GE HealthCare Technologies Inc., Chicago, IL, USA).

The chest CT scans were performed using either a 128-slice (SOMATOM Definition Flash, SIEMENS Healthineers AG, Forchheim, Germany) or a 64-slice CT scanner (SOMATOM go.Top, SIEMENS Healthineers AG, Forchheim, Germany). The images were acquired during a single breath-hold from the lung bases to the apices. If the examination was requested to confirm (or eliminate) the suspected diagnosis of COVID-19 pneumonia, the scans were performed without the administration of i. v. contrast media. Yet, some of the patients who received their CT examinations for other clinical indications, e.g., suspected pulmonary embolism or oncologic imaging, received 40 to 80 mL of an iodine-containing contrast agent (ACCUPAQUE^TM^-300 or -350, GE HealthCare Buchler GmbH & Co. KG, Braunschweig, Germany) i. v.

The CT scans were acquired using an automatic exposure control system, both for the tube voltage and the tube current. From the raw data, the slices were reconstructed with a sharp (pulmonary) and a softer (mediastinal) kernel. The images in the lung kernel were reconstructed in different slice thicknesses (0.8, 1, 2, 3, or 5 mm).

The CT images were analysed by three different readers: a radiology specialist (senior physician), an experienced assistant physician, and a medical student. The readers evaluated independently if specific pathological features of the lung parenchyma were present that are typical of COVID-19, namely ground-glass opacities (GGO), consolidations, and a crazy-paving pattern. Other abnormalities that have also been described in connection with COVID-19, e.g., nodules or a halo sign, were also recorded as parenchymal changes. All readers were blinded with regard to the clinical data (laboratory values, blood gas analyses, intensive medical care, etc.) of the patients and for each other’s assessments.

No formal calibration session or consensus reading was conducted prior to the actual image assessment, but the medical student received several hours of focused training in advance, which enabled him to differentiate between the individual lung lobes, to recognise the different pulmonary pathologies to be evaluated, and to quantify them based on their extent. The two radiologists have so many years of experience in chest CT (14 and 6 years, respectively) that they were not trained separately but merely informed about which pathologies (according to the Fleischner Society glossary) they should assess and how the CT score should be applied. Since the evaluation of the CT data began in 2023, the definitions of the radiological terms are based on the 2008 version of the Fleischner Society glossary [8].

According to the semi-quantitative score proposed by Pan et al., the proportion of parenchyma affected by pathological changes was recorded individually for each of the five lobes of the lung. A subjective, visual score from 0 to 5 was assigned for each lobe (Table 1):

The scores of all lobes were then added to a total score between 0 and 25.

**Figure 1:**
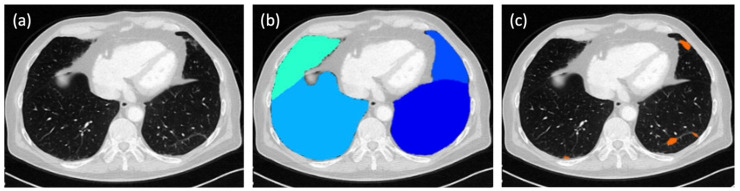
Fully automated evaluation of a chest CT scan by an AI-based software tool (ADVANCE chest CT, contextflow GmbH, Vienna, Austria): (**a**) CT image without any software overlays; (**b**) automated segmentation of the lung lobes (turquoise, middle lobe; light blue, right lower lobe; blue, lingula; dark blue, left lower lobe); (**c**) AI-generated detection of ground-glass opacities (GGO) in the left lower lobe, the lingula, and the right lower lobe, marked in orange.

In parallel to the evaluation by the three human readers, the score for each patient was calculated fully automatically by a dedicated, AI-based software tool (ADVANCE chest CT, contextflow GmbH, Vienna, Austria; Figure 1). In this way, four different total scores were available for each patient for comparison.

The system applies a deep-learning-based algorithm to lung kernel images to automatically segment and quantify parenchymal abnormalities. No manual corrections were made to the AI results. According to the manufacturer, the system was trained on a multicentric CT data set; however, the exact training details and algorithm architecture remain proprietary.

All statistical analyses and visualisations were performed using R (version 4.x; R Core Team, 2024) [9]. The analyses included descriptive statistics, inter-rater agreement measures, and hypothesis testing. Core functions from the stats package were used for ANOVA and Wilcoxon tests. Inter-rater reliability was calculated using the psych [10] and irr [11] packages, while the DescTools package supported additional descriptive procedures [12]. Visualisations were created using ggplot2 [13], and data handling was conducted using functions from the tidyverse collection [14].

A total score for each individual CT was calculated for every reader by summing up the scores of all five lung lobes, resulting in a score ranging from 0 (no abnormalities) to 5 (>75% conspicuity in all lobes). These total scores were statistically compared across all human readers (student, physician, senior physician) and the AI-based software. The normality of the score distributions was assessed using the Shapiro–Wilk test. All reader-specific score distributions deviated significantly from a normal distribution (*p* < 0.001). Therefore, a non-parametric Kruskal–Wallis test [10] was conducted to compare total scores between the four reader groups.

All assigned scores were additionally compiled into frequency distributions to descriptively explore potential differences in rating behaviour between the readers. Frequencies were visualised to support the interpretation.

To assess inter-reader agreement, pairwise comparisons were performed using weighted Cohen’s kappa (κ), which accounts for the ordinal nature of the data. Furthermore, the mean κ value of all human reader pairs was calculated and compared to the values obtained from comparisons involving the AI-based software.

The intraclass correlation coefficient [ICC(2,1)] was calculated using a two-way random effects model to quantify the overall agreement of total scores across all four readers.

The potential influence of slice thickness on scoring behaviour was analysed at two levels: inter-reader agreement and reader-assigned total scores. For the former, pairwise κ values were grouped by slice thickness (0.8 & 1 combined, 2, 3, and 5 mm) and evaluated using one-way ANOVA via the aov() function [11]. For the latter, the senior physician’s total scores were grouped by slice thickness and analysed analogously.

The effect of i. v. contrast administration was assessed by comparing total scores from scans with and without contrast medium. A Wilcoxon rank sum test [9] was performed to test for significant differences between the two groups, using a 5% significance level.

Finally, mean scores were calculated for each individual lung lobe—including the middle lobe, both upper lobes, and both lower lobes—across all reader groups to explore regional scoring tendencies in relation to reader experience and algorithmic support.

## 3. Results

**Figure 2:**
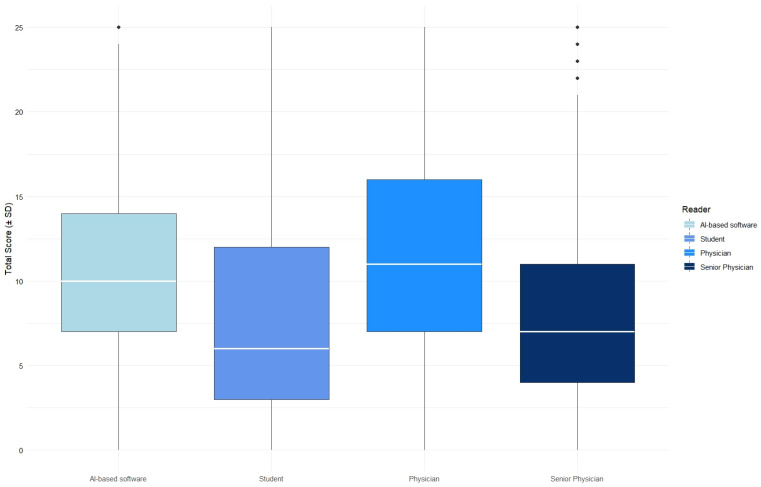
Comparison of the total scores of all four readers (senior physician, physician, student, and AI); one-way analysis of variance (Kruskal–Wallis test) (*p* < 0.001).

The total score was initially calculated for each human reader and for the AI-based software individually. The radiology specialist (senior physician) achieved a mean score of 8.32 ± 5.92, the assistant physician (physician) a score of 11.49 ± 6.26, the medical student (student) a score of 8.25 ± 6.74, and the software (AI) a score of 10.44 ± 5.10 (Figure 2 and Table 2). The results of the different readers differed significantly (*p* < 0.001).

**Figure 3:**
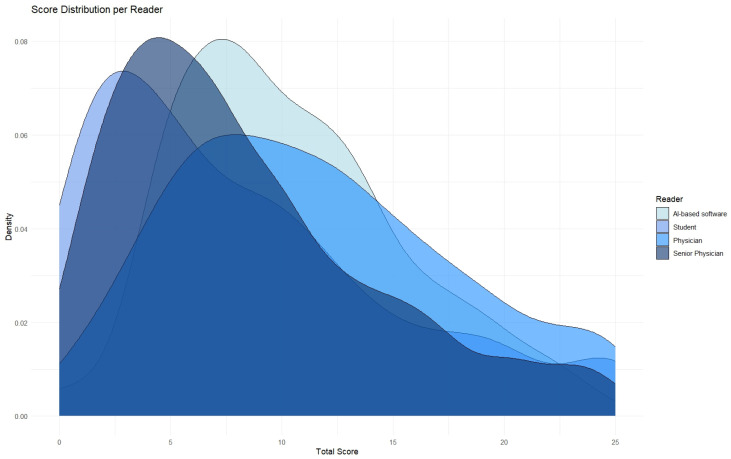
Kernel density plot showing the distribution of total Pan-scores across all reader groups (AI-based software, student, physician, and senior physician).

The distribution of the different scores as evaluated by the three readers and the AI are displayed in Figure 3. Notable differences were observed in the frequency of score assignments. The senior physician and the AI-based software most frequently assigned a score of 1 (senior physician, 39.1%; AI, 32.4%), followed by score 2 (senior physician, 21.6%; AI, 30.1%). In contrast, the physician and the student more often assigned higher scores. The physician most frequently chose scores 2 (27.5%) and 3 (21.4%), while the student assigned scores of 1 (34.0%), 0 (29.3%), and 2 (19.2%) most often. Scores 4 and 5 were more frequently observed in human ratings than in the AI’s output: for example, the physician assigned score 4 in 13.9 and score 5 in 10.4% of cases, compared to only 11.7 and 3.4%, respectively, for the AI. This indicates a systematic variation in the scoring behaviour, depending on reader experience and the use of automated analysis.

**Figure 4:**
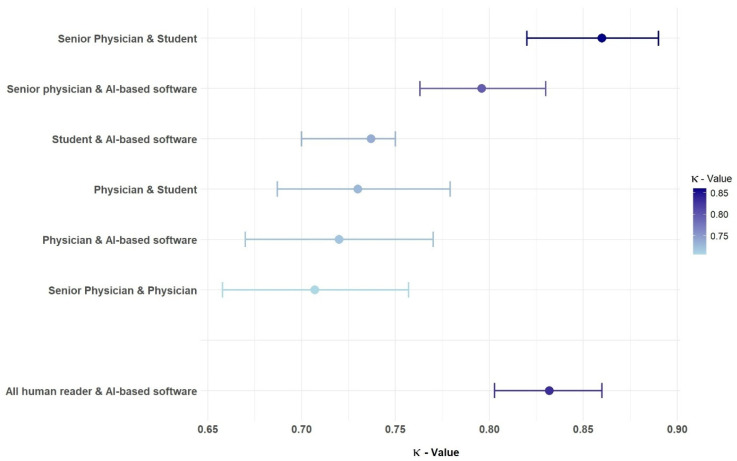
Illustration of the pairwise inter-rater agreement between all human readers and the AI, expressed as weighted κ values with corresponding 95% confidence intervals.

The inter-rater agreement was quantified by determining the κ values (Figure 4). The greatest agreement (κ = 0.86) was observed between the senior physician and the student, whose scores did not differ significantly (*p* = 0.99), whereas the senior physician and the physician agreed the least (κ = 0.71). When comparing the human readers with the AI, the total score of the senior physician showed the greatest agreement (κ = 0.80) with the score evaluated by the software.

Beyond pairwise agreement (κ), the overall agreement in total score ratings across all four readers was assessed using the intraclass correlation coefficient [ICC(2,1)]. The calculated ICC was 0.85 (95% confidence interval: 0.81–0.88), indicating excellent consistency across readers.

With all κ values > 0.7, the overall inter-observer variability between the readers was low, and they showed substantial agreement in their scores.

In order to compare the human readers with the AI, the average total score across the three readers was calculated (9.35 ± 6.03). This score differed significantly from the AI’s average score (*p* < 0.001).

**Figure 5:**
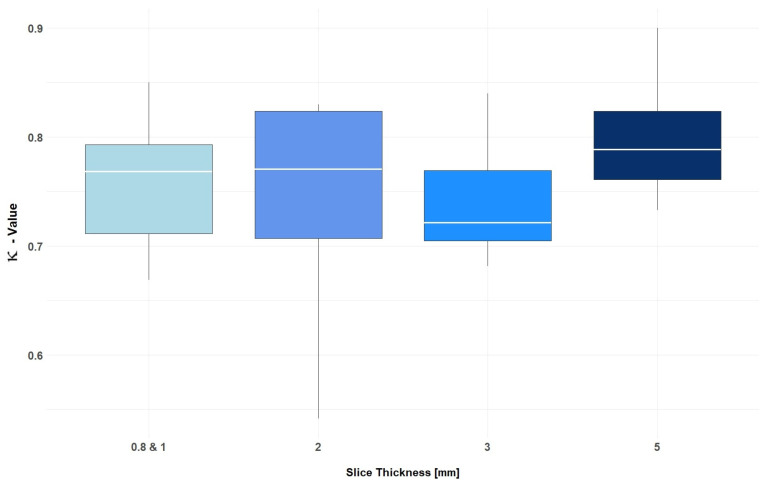
Illustration of the inter-rater agreement for each individual slice thickness, expressed as weighted κ values with corresponding 95% confidence intervals. The mean κ values for the different slice thicknesses were 0.76 for 0.8 & 1 mm combined, 0.74 for 2 mm, 0.74 for 3 mm, and 0.80 for 5 mm (one-way ANOVA, *p* = 0.57).

To assess whether varying slice thicknesses of the CT images—particularly the distinct differences between 0.8 and 5 mm—had an impact on scoring, the inter-rater agreement was re-evaluated separately for each slice thickness (Figure 5). Notably, more than half of the images were reconstructed with a thickness of 3 mm. Slices with a thickness of 0.8 and 1 mm showed a mean κ value of 0.76, a slice thickness of 2 mm resulted in a mean κ value of 0.74, 3 mm slices showed a mean value of 0.74, and 5 mm slices reached a mean κ value of 0.80; these values did not differ significantly (*p* = 0.57).

**Figure 6:**
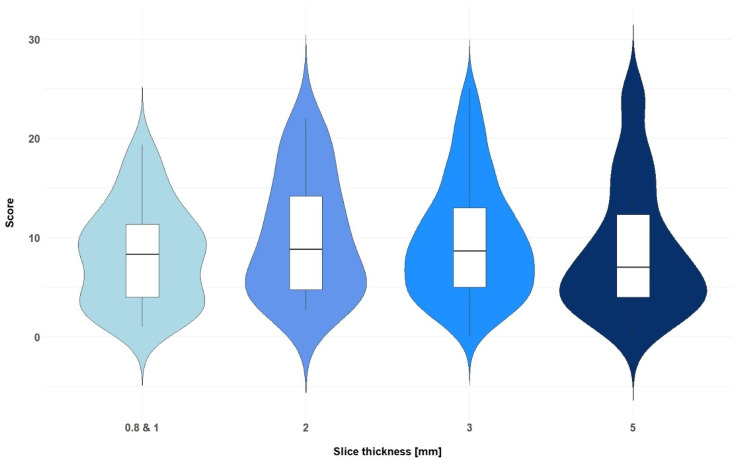
Distribution of the total scores of the senior physician across the different slice thicknesses with 95% confidence intervals (one-way ANOVA, *p* = 0.66).

Figure 6 exemplarily shows the distribution of the senior physician’s total scores across the different slice thicknesses, which showed no significant difference (*p* = 0.66).

**Figure 7:**
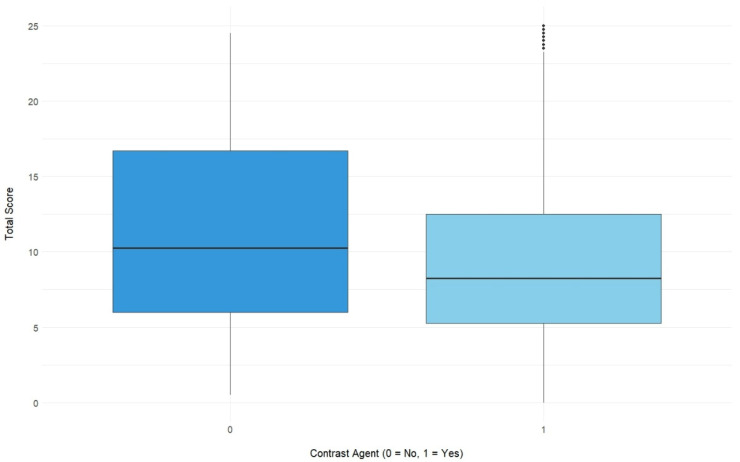
Illustration of the inter-rater agreement for images without or with the i. v. administration of a contrast medium, expressed as total scores with corresponding 95% confidence intervals (Wilcoxon rank sum test: *p* = 0.002).

However, there was a statistically significant difference (*p* = 0.002) between the total scores evaluated based on CT images acquired without or after the i. v. administration of a contrast medium (Figure 7).

**Figure 8:**
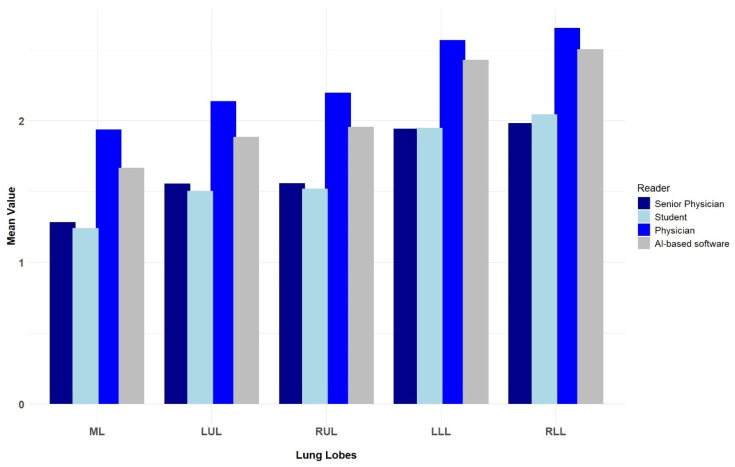
Mean scores of the individual lung lobes. ML, middle lobe; LUL, left upper lobe; RUL, right upper lobe; LLL, left lower lobe; RLL, right lower lobe.

The lower lobes (the right more than the left) were most frequently and/or most severely affected by COVID-associated changes, as indicated by all three human readers and the AI-based software (Figure 8).

## 4. Discussion

The objective of this study was to determine the practicability and diagnostic accuracy of the semi-quantitative CT score proposed by Pan et al. for the assessment of the extent of pulmonary changes associated with COVID-19. The performance of the scoring system was evaluated under varying circumstances, including different image acquisition parameters and experiences of the reader. For this purpose, CT images of COVID-positive patients were analysed, which were acquired no more than three days before or after the positive PCR test. The sample size of our study (*n* = 569) is comparable to that of Almalki et al. (*n* = 630), who similarly assessed inter-rater agreement in chest CT scoring of COVID-19 patients across different reader experience levels [15].

In the survey of the total score, the inter-rater agreement both between the human readers and with the AI was substantially high (κ > 0.7). Although the mean scores ranged from 8.25 ± 5.92 to 11.49 ± 6.26 and thus differed significantly in most cases, the overall agreement was high, which was partly due to the large standard deviations. In addition, both the lowest and the highest scores indicated a moderate extent of pulmonary changes, meaning that the different scores would not have had any clinical consequences in terms of different treatment strategies, especially since these are mainly determined by significant other factors (oxygen saturation, dyspnoea, etc.) [16].

The radiology specialist showed the highest level of agreement with the medical student, followed by the agreement with the AI. These two κ values were higher than those of all other readers compared with each other. Interestingly, the lowest agreement was found between the radiology specialist and the assistant physician. The fact that the assistant physician also agreed less with the medical student and with the AI than the other readers is most likely an expression of the subjective nature of this purely visual score, which depends heavily on how well someone can estimate.

When looking at the scores assigned to each lung lobe individually, three of the four readers agreed that they most frequently assigned the score 1 (<5% involvement); only the assistant physician awarded the score 2 (5–25% involvement) most frequently. Respectively, the least frequently awarded score with three of the four readers was a score of 5 (>75% involvement); only the medical student chose the score 4 (50–75% involvement) least frequently.

Considering the extent of pulmonary changes for each individual lung lobe, all four readers reported that the right lower lobe was the most severely affected, followed by the left lower lobe. This is consistent with the results of a meta-analysis in which the pooled prevalence values of COVID-related changes in the right and left lower lobes were 72.2 and 73.1%, respectively [17].

The reconstructed slice thickness of the CT images had no impact on the score, meaning that the score can be reliably used with different image reconstructions (i.e., reconstructed slice thicknesses).

However, the score was significantly higher in patients who did not receive i. v. contrast media prior to their CT scan. Since the examinations that were performed to confirm (or eliminate) the suspected diagnosis of COVID-19 pneumonia (i.e., in patients who showed symptoms of COVID-19 pneumonia) were acquired without the administration of i. v. contrast media, the amount of heavily affected patients in this group is likely to be greater than in the group of patients who received i. v. contrast media, as this group also comprised patients who did not have any pulmonary symptoms, but had their CT examination due to other medical issues. Since the scores did not differ to an extent that would be clinically relevant, the Pan-score can apparently also be reliably determined based on contrast-enhanced CT imaging, although it is recommended to perform an unenhanced chest CT if pulmonary involvement of COVID-19 is clinically suspected [18,19].

One limitation of this study is its retrospective character. Furthermore, the actual extent of the pulmonary changes is unknown because this could only be reliably determined by an autopsy. In order to tackle this problem, our working group is already in the process of analysing the clinical outcomes of the affected patients, including available follow-up CT scans, various laboratory values, blood gas analyses, and spirometry results, and their correlation with the CT scores obtained.

Another limitation is the fact that only one AI-based software solution was used. However, this was a deliberate decision, as the focus was not on evaluating the performance of different software tools, but on comparing the results of human and artificial readers. We therefore only used the software that is commercially available to our institute.

The absence of a standardised CT protocol (in terms of slice thicknesses) at the time the evaluated CT examinations were acquired, could also be interpreted as a restriction. In truth, however, it has enabled us, in this way, to show that the slice thickness had no influence on the accuracy of the Pan-score.

Although the clinical relevance of evaluating patients with COVID-19 has decreased due to the declining number of cases of severe disease progression, the infection remains endemic, and further waves of spread are expected to occur [1]. In addition, the evaluation of semi-quantitative CT scores remains important, as they can be adapted for the assessment of other pulmonary infectious disorders in the future, and the automated analysis of various pulmonary diseases will remain a topical issue in view of the rapid further development of AI-based software solutions.

## 5. Conclusions

The evaluated semi-quantitative CT score is a simple but reliable diagnostic tool that enables radiologists of different experience to quickly assess the severity of patients suffering from COVID-19 pneumonia. Although the scores of the individual readers differed significantly in some cases, the respective assessments all indicated the same severity level of COVID-19 pneumonia, meaning that the differences would have had no influence on clinical management. The high inter-rater agreement both between differently experienced human readers and a dedicated AI-based software tool emphasises the reproducibility of the score and demonstrates the possibility to determine the extent of pulmonary involvement both subjectively and fully automatically.

## Figures and Tables

**Table 1:** Semi-quantitative chest CT score, according to Pan et al. [7], to evaluate the proportion of the affected lung parenchyma by pathological changes due to COVID-19 pneumonia.

Score	Extent of Involvement of Every Single Lung Lobe
0	No involvement
1	<5%
2	5–25%
3	26–49%
4	50–75%
5	>75%

**Table 2:** Total scores of the four different readers (senior physician, physician, student, and AI), displayed as mean ± standard deviation.

Reader	Total Score
Senior physician	8.32 ± 5.92
Physician	11.49 ± 6.26
Student	8.25 ± 6.74
AI	10.44 ± 5.10

## Data Availability

The data sets (chest CT images) presented in this article are not readily available due to privacy and ethical restrictions.

## Abbreviations

The following abbreviations are used in this manuscript:

AI	Artificial intelligence
ANOVA	Analysis of variance
COVID-19	Coronavirus disease 2019
CT	Computed tomography
GGO	Ground-glass opacity
ICC	Intraclass correlation coefficient
i. v.	Intravenous
LLL	Left lower lobe
LUL	Left upper lobe
ML	Middle lobe
PACS	Picture archiving and communication system
PCR	Polymerase chain reaction
RIS	Radiological information system
RLL	Right lower lobe
RUL	Right upper lobe
SARS-CoV-2	Severe acute respiratory syndrome coronavirus 2
WHO	World Health Organisation

## References

[1] Robert Koch-Institut, COVID-19 RKI-Ratgeber. https://www.rki.de/DE/Aktuelles/Publikationen/RKI-Ratgeber/Ratgeber/Ratgeber_COVID-19.html?nn=16911046.

[2] Vogel-Claussen J., Ley-Zaporozhan J., Agarwal P. (2020). Recommendations of the thoracic imaging section of the German Radiological Society of clinical application of chest imaging and structured CT reporting in the COVID-19 pandemic. Rofo.

[3] Ye Z., Zhang Y., Wang Y. (2020). Chest CT manifestations of new coronavirus disease 2019 (COVID-19): A pictorial review. Eur. Radiol..

[4] Li J., Yan R., Zhai Y. (2021). Chest CT findings in patients with coronavirus disease 2019 (COVID-19): A comprehensive review. Diagn. Interv. Radiol..

[5] Szabó M., Kardos Z., Kostyál L. (2023). The importance of chest CT severity score and lung CT patterns in risk assessment in COVID-19-associated pneumonia: A comparative study. Front. Med..

[6] Francone M., Iafrate F., Masci G.M. (2020). Chest CT score in COVID-19 patients: Correlation with disease severity and short-term prognosis. Eur. Radiol..

[7] Pan F., Ye T., Sun P. (2020). Time course of lung changes at chest CT during recovery from coronavirus disease 2019 (COVID-19). Radiology.

[8] Hansell D.M., Bankier A.A., MacMahon H. (2008). Fleischner Society: Glossary of terms for thoracic imaging. Radiology.

[9] R Core Team, the R Foundation. https://www.r-project.org.

[10] Revelle W. psych: Procedures for Psychological, Psychometric, and Personality Research. https://cran.r-project.org/web/packages/psych/index.html.

[11] Gamer M., Lemon J., Fellows I. irr: Various Coefficients of Interrater Reliability and Agreement. https://cran.r-project.org/web/packages/irr/index.html.

[12] Signorell A., Aho K., Alfons A. DescTools: Tools for Descriptive Statistics. https://cran.r-project.org/web/packages/DescTools/index.html.

[13] Wickham H., Chang W., Henry L. ggplot2. https://ggplot2.tidyverse.org/.

[14] Wickham H., Averick M., Bryan J. (2019). Welcome to the Tidyverse. J. Open Source Softw..

[15] Almalki Y.E., Basha M.A.A., Metwally M.I. (2023). Inter-observer variability in the analysis of CO-RADS classification for COVID-19 patients. Trop. Med. Infect. Dis..

[16] World Health Organization Therapeutics and COVID-19: Living Guideline (v14.1). https://app.magicapp.org/#/guideline/nBkO1E.

[17] Adams H.J.A., Kwee T.C., Yakar D. (2020). Chest CT imaging signature of coronavirus disease 2019 infection: In pursuit of the scientific evidence. Chest.

[18] Rodrigues J.C.L., Hare S.S., Edey A. (2020). An update on COVID-19 for the radiologist–a British Society of Thoracic Imaging statement. Clin. Radiol..

[19] Kwee T.C., Kwee R.M. (2020). Chest CT in COVID-19: What the radiologist needs to know. Radiographics.

